# Sitosterolemia: A Case Report and a Concise Literature Review

**DOI:** 10.1155/2023/4451595

**Published:** 2023-03-08

**Authors:** Moeber M. Mahzari

**Affiliations:** ^1^College of Medicine, King Saud bin Abdulaziz University for Health Sciences, Riyadh, Saudi Arabia; ^2^King Abdullah International Medical Research Center, Riyadh, Saudi Arabia; ^3^Ministry of the National Guard-Health Affairs, Riyadh, Saudi Arabia

## Abstract

**Background:**

Sitosterolemia is a relatively rare metabolism lipid disorder, with about 110 cases worldwide and only a few known cases from the Middle East. Sitosterolemia is characterized by excessive uptake of phytosterols and their deposition in various tissues, leading to complications. Mutations in the ABCG5 and ABCG8 genes are associated with pathological changes in sitosterolemia. *Case Presentation*. An adult patient from Saudi Arabia with dyslipidemia who did not respond to statin therapy. Based on genetic testing, the patient was eventually diagnosed with sitosterolemia. Ezetimibe significantly improved his cholesterol levels.

**Conclusion:**

The diagnosis of sitosterolemia is confirmed by the detection of high-phytosterol levels and pathological mutation in the ABCG5 and ABCG8 genes. Treatment of sitosterolemia is based on dietary changes and drugs to inhibit cholesterol absorption, such as ezetimibe.

## 1. Introduction

Sitosterolemia was first described by Battacharyya and Connor in 1974 as a lipid disorder characterized by tendon and tuberous xanthomas causing premature coronary atherosclerosis [1].

Autosomal mutations in the ABCG5 and ABCG8 genes are widely recognized as the cause of sitosterolemia. Patients with homozygous or combined heterozygous mutations in the ABCG5 and/or ABCG8 genes experience increased absorption of dietary fats in conjunction with decreased excretion via the biliary system [2]. This results in high levels of plant sterols and LDL cholesterol in the circulation [2]. The phytosterols are subsequently stored and deposited in various tissues, particularly in the vasculature, leading to atherosclerosis [3]. In addition, one-third of patients with sitosterolemia experience hematologic disorders such as stomatocytosis, large platelets, and hemolytic anemia due to the deleterious effect of excessive phytosterols on the formation and function of red blood cells and platelets [4].

The diagnosis of sitosterolemia is difficult because the elevation of LDL cholesterol is usually similar to that seen in the mixed type and familial hypercholesterolemia. As with the familial hypercholesterolemia, the diagnosis of sitosterolemia is often suspected in patients with dyslipidemia at a young age, especially in patients who respond poorly to statins. The diagnosis is confirmed by detection of elevated dietary sterols in plasma. However, a routine lipid profile does not reveal the source of elevated cholesterol in the circulation. Therefore, more accurate measurement of different types of cholesterol with gas chromatography-mass spectrometry is usually needed to confirm high levels of plant sterols. Moreover, the diagnosis is confirmed by the detection of homozygous or double heterozygous mutations in the ABCG5 and ABCG8 genes [4].

Patients with sitosterolemia usually respond poorly to statins, which have minimal effects on LDL-C levels in such patients [5]. On the contrary, they respond well to dietary measures and drugs that inhibit cholesterol absorption in the intestine such as ezetimibe. Early diagnosis of sitosterolemia is crucial to timely institute proper management and prevent complications [6].

Sitosterolemia is a rare inherited lipid disorder. The prevalence is estimated to be 1 in 360,000–2.6 million [7]. However, case reports of sitosterolemia have been published from multiple areas around the world [8]. To date, no case of adult sitosterolemia has been reported from Saudi Arabia or the Middle East region. The clinical course of a Saudi patient with sitosterolemia including diagnosis and treatment is presented.

## 2. Case Report

**Figure 1:**
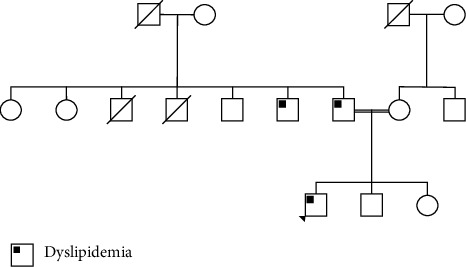
The patient's family tree.

A 30-year-old male patient was evaluated for high LDL cholesterol three years after initial diagnosis of dyslipidemia. According a routine blood examination, the patient was found to have high cholesterol (Table 1). He then took rosuvastatin 10 mg, which had no significant effect on cholesterol levels. Rosuvastatin was then discontinued after several months. The patient also has a history of chronic mild thrombocytopenia with megakaryocytes, which was treated conservatively. The patient has a family history of dyslipidemia in his father and paternal uncle. There is a consanguineous relationship between the patient's parents, who are cousins (Figure 1). The patient is not known to have any other medical problems and exercises regularly. The patient denied history of chest pain or limitation in his exercise capacity. Moreover, he never required any investigations for cardiovascular diseases. On examination at the clinic, a small xanthoma was noted at the elbow tendon, but no xanthelasma or organomegaly. Repeated blood tests showed elevated LDL cholesterol (Table 1). He was then prescribed rosuvastatin 20 mg once daily because it was thought to be mixed hypercholesterolemia or heterozygous familial hypercholesterolemia. However, the LDL cholesterol level remained almost unchanged 3 months later. Genetic testing was then performed to further guide the patient's treatment. Genetic testing provided by Centogene NV (Germany) was performed, which included 46 genes associated with hereditary hypercholesterolemia, including the LDLR, Apo-B, PCSK9, and ABCG genes. The patient was found to have a homozygous missense mutation in the ABCG8 gene (c.1715T > C, p.(Leu572Pro)). This mutation has been previously described as a cause of sitosterolemia [9]. He was subsequently treated with ezetimibe 10 mg daily and rosuvastatin was discontinued. Approximately 3 months after starting ezetimibe treatment, there was a marked improvement in his LDL cholesterol (Table 1). The measurement of plant sterols was not performed because they were not available. The patient's platelet count and platelet volume varied between normal and slightly abnormal.

## 3. Discussion

Sitosterolemia is a rare disorder characterized by high plant sterols and LDL cholesterol. Patients with sitosterolemia tend to develop several complications. These complications are primarily due to the deposition of plant sterols in different tissues. Patients with sitosterolemia show a variety of manifestations such as xanthomas at different sites. Moreover, deposition of phytosterols in the cardiovascular system leads to complications such as coronary and carotid artery stenosis and, less commonly, valvular heart disease. Early diagnosis is critical to initiate early intervention and prevent future life-threatening complications [6, 10]. Fortunately, the patient reported here was discovered before complications arose.

Overall, sitosterolemia is quite rare with only about 110 reported cases worldwide [11]. General cases of sitosterolemia are reported more frequently in children than in adults and as early as 3 months of age [12]. There are few cases of sitosterolemia in patients of Arab descent. Taher et al. reported a child from Saudi Arabia, and Bardawil et al. reported a family from Tunisia with sitosterolemia [13, 14]. To my knowledge, the patient reported here is the first adult patient with sitosterolemia from Saudi Arabia and the Middle East region. However, sitosterolemia is likely underdiagnosed in this region. In the Middle East in general and Saudi Arabia in particular, familial diseases are relatively common due to the founder effect of consanguineous marriages. For example, the predicted prevalence of familial hypercholesterolemia in the Middle East region is 1 : 200, which is higher than expected. This higher prevalence is attributed to the founder effect [15]. On this note, sitosterolemia is also expected to be relatively more common than reported in the Middle East region. The low prevalence of sitosterolemia in the region with only one case report is likely due to under diagnosis of the condition.

The majority of patients with sitosterolemia are diagnosed in the setting of dyslipidemia with high LDL cholesterol with or without complications such as large xanthomas or cardiovascular complications.

The diagnosis of sitosterolemia is based on confirmation of high levels of dietary sterols in plasma or confirmation of homozygous or double heterozygous mutation in ABCG5 and/or ABCG8 [4]. Measurement of phytosterols requires specialized processing that is not readily available. The phytosterol was not measured in the patient because it was not available. Molecular diagnosis of lipid disorders is a well-established practice of precision medicine that is helpful in confirming the diagnosis and guiding treatment [16].

A genetic test was performed in the patient, who was suspected to have mutations associated with familial hypercholesterolemia. However, the test revealed a normal LDL receptor, PCSK9, and Apo-B gene panel, whereas a homozygous missense mutation in the ABCG8 gene was confirmed. This mutation in the ABCG8 gene has been associated with sitosterolemia [9]. In addition, this mutation has been reported to cause hematological derangements such as hereditary stomatocytosis and bone marrow failure [17]. Retrospectively, the patient's thrombocytopenia and large platelets were associated with the diagnosis of sitosterolemia.

It seems that ABCG5 gene mutations are more common in patients of Asian descent, especially in Chinese and Japanese patients, while mutations in the ABCG8 gene are more common in other populations [12, 18]. It is recommended to obtain cascade genetic testing of such patient. However, that is not always possible. In the reported patient, cascade genetic testing was not performed in his family because they were not able to attend the clinic.

When treating patients with sitosterolemia, it is critical to confirm the diagnosis. Treatment of dyslipidemia, generally, involves dietary changes and lipid-lowering medications. The general advice for patients with dyslipidemia is to eat a healthy plant-based or Mediterranean diet rich in fruit, vegetables, and nuts [19]. However, such a diet may have a paradoxical effect on the lipid profile of patients with sitosterolemia. On the contrary, patients with sitosterolemia must avoid plant sources of fat [20]. Although the reported patient led a healthy lifestyle, his lipid profile did not improve. This was probably due to the fact that the patient followed the general dietary recommendations for dyslipidemia and focused on plant sources of fat, which resulted in an increased intake of phytosterols. Almost all patients with sitosterolemia respond poorly to statin therapy, but their lipid profile improves with cholesterol absorption inhibitors such as ezetimibe [20]. Similar to the reported cases, high-intensity statin had only a marginal effect on the patient. Based on the result of the genetic screen, the statin was discontinued and ezetimibe was started, resulting in a dramatic improvement in the lipid profile. Indeed, it appears that the dramatic response to ezetimibe in patients with poor response to statin therapy may suggest the possibility of sitosterolemia [6].

## 4. Conclusion

Sitosterolemia is a relatively rare lipid metabolism disorder but is likely underdiagnosed, especially in populations where a founder effect is expected. To my knowledge, the patient reported here is the first adult patient with sitosterolemia from the Middle East/Saudi Arabia. The diagnosis of sitosterolemia was confirmed by molecular testing, which then led to appropriate treatment with ezetimibe.

## Figures and Tables

**Table 1:** Patient's laboratory values over time.

Exam. names	Reference values	3 years and 6 months after diagnosis (on ezetimibe 10 mg only for 3 months)	3 years and 3 months after diagnosis (on rosuvastatin 20 mg for 3 months)	3 years after diagnosis (no treatment)	1 year after diagnosis (no treatment)	6 months after diagnosis (on rosuvastatin 10 mg)	Up on diagnosis
Hemoglobin	135 ∼ 180 gm/L	130		140	135	138	135
Platelet	150 ∼ 400 × 10^9^/L	174		142	158	190	168
Mean platelet volume	7.4 ∼ 10.4 fL	12.8		14.3	12.8	9.7	12
HDL-C	Negative risk factor for heart disease is ≥1.55 mmol/L	1.35	1.47	1.32	1.21	1.31	1.36
LDL-C	Optimal <2.59 mmol/L	2.41	4.37	5.02	6.03	4.48	6.03
Total cholesterol	Desirable <5.18 mmol/L	4.19	6.1	6.91	7.62	6.29	7.75
Triglyceride	Optimal:<1.70 mmol/L	0.47	0.68	0.84		0.7	0.88
Fasting glucose	4.1 ∼ 5.6 mmol/L	5.1				5	4.6

## Data Availability

The patient data used to support the findings of this study are included within the article.
